# Elimination of Omega-1,2 Gliadins From Bread Wheat (*Triticum aestivum*) Flour: Effects on Immunogenic Potential and End-Use Quality

**DOI:** 10.3389/fpls.2019.00580

**Published:** 2019-05-09

**Authors:** Susan B. Altenbach, Han-Chang Chang, Xuechen B. Yu, Bradford W. Seabourn, Peter H. Green, Armin Alaedini

**Affiliations:** ^1^Western Regional Research Center, United States Department of Agriculture-Agricultural Research Service, Albany, CA, United States; ^2^Department of Medicine, Columbia University, New York, NY, United States; ^3^Institute of Human Nutrition, Columbia University, New York, NY, United States; ^4^Hard Winter Wheat Quality Laboratory, Center for Grain and Animal Health Research, United States Department of Agriculture-Agricultural Research Service, Manhattan, KS, United States; ^5^Celiac Disease Center, Columbia University, New York, NY, United States; ^6^Department of Medicine, New York Medical College, Valhalla, NY, United States

**Keywords:** celiac disease, wheat allergy, gliadins, gluten proteins, gluten-related disorders, proteomics, wheat flour quality

## Abstract

The omega-1,2 gliadins are a group of wheat gluten proteins that contain immunodominant epitopes for celiac disease (CD) and also have been associated with food allergies. To reduce the levels of these proteins in the flour, bread wheat (*Triticum aestivum* cv. Butte 86) was genetically transformed with an RNA interference plasmid that targeted a 141 bp region at the 5′ end of an omega-1,2 gliadin gene. Flour proteins from two transgenic lines were analyzed in detail by quantitative two-dimensional gel electrophoresis and tandem mass spectrometry. In one line, the omega-1,2 gliadins were missing with few other changes in the proteome. In the other line, striking changes in the proteome were observed and nearly all gliadins and low molecular weight glutenin subunits (LMW-GS) were absent. High molecular weight glutenin subunits (HMW-GS) increased in this line and those that showed the largest increases had molecular weights slightly less than those in the non-transgenic, possibly due to post-translational processing. In addition, there were increases in non-gluten proteins such as triticins, purinins, globulins, serpins, and alpha-amylase/protease inhibitors. Reactivity of flour proteins with serum IgG and IgA antibodies from a cohort of CD patients was reduced significantly in both transgenic lines. Both mixing time and tolerance were improved in the line without omega-1,2 gliadins while mixing properties were diminished in the line missing most gluten proteins. The data suggest that biotechnology approaches may be used to create wheat lines with reduced immunogenic potential in the context of gluten sensitivity without compromising end-use quality.

## Introduction

Wheat is a major food crop grown throughout the world that is used in a wide range of different food products because of the unique viscoelastic properties of the flour. These properties are conferred by the gluten proteins, a complex group of proteins that account for about 70–80% of the total flour protein and are unusual in that they contain large regions of repetitive sequences with high proportions of glutamine and proline. The gluten proteins are divided into two major groups referred to as gliadins and glutenins. The gliadins are present in the flour as monomers and consist of alpha, gamma, delta and omega types, each with distinct structures, N-terminal sequences and repetitive motifs. These proteins confer extensibility to wheat flour dough. In comparison, the glutenins are present as large insoluble polymers made up of two types of proteins that are linked by disulfide bonds. These proteins are referred to as HMW-GS and LMW-GS. The glutenin polymers contribute elasticity to wheat flour dough. Ultimately, the composition of the gluten proteins in the flour, determined by both the genetics of the plant and the growth environment, is critical for end-use quality.

Each gluten protein group is encoded by many similar genes and there is considerable allelic variation among different wheat cultivars. Most bread wheat cultivars contain only six HMW-GS genes, while the numbers of gliadin and LMW-GS genes are much higher. Only recently with the availability of a high-quality wheat genome sequence from the reference wheat Chinese Spring (Zimin et al., 2017; International Wheat Genome Sequencing Consortium [IWGSC], 2018) has it been possible to determine accurately the complexity of these gene families in a single wheat cultivar. Indeed, a complete set of genes assembled and annotated by Huo et al. (2018a,b) from Chinese Spring included 102 genes of which 47 were alpha gliadins, 14 were gamma gliadins, five were delta gliadins, 19 were omega gliadins and 17 were LMW-GS. Of these, 26 alpha, 11 gamma, two delta, five omega gliadin and 10 LMW-GS encoded full-length proteins, while the remaining genes were either partial sequences or pseudogenes.

While the complexity of the wheat gluten proteins and their genes makes wheat research challenging, this is compounded by the fact that some of the same proteins that determine the commercial value of the flour also trigger human health conditions, including CD and IgE-mediated food allergies (Scherf et al., 2016, for review). T-cell epitopes that are relevant for CD have been identified in all of the major gluten protein groups. A list compiled by Sollid et al. (2012) includes five distinct epitopes from alpha gliadins, ten from gamma gliadins, two from omega gliadins, two from LMW-GS and one from HMW-GS. Of the gluten proteins, alpha gliadins have been thought to harbor some of the most important epitopes and a protease-resistant 33-mer peptide found in some alpha gliadins has been shown to be particularly toxic (Shan et al., 2002). Interestingly, the 33-mer peptide consists of six overlapping CD epitopes. The numbers of epitopes in individual gluten proteins also can vary considerably within a cultivar. For example, of the 26 alpha gliadins from Chinese Spring, only one protein contains the 33-mer peptide while nine proteins, all encoded by the B genome, do not contain any of the previously described CD epitopes. The rest contain from one to nine CD epitopes (Huo et al., 2018a). Certain omega gliadins also are immunodominant in CD. In fact, Tye-Din et al. (2010) reported that epitopes found in the omega-1,2 gliadins have a level of immunogenicity similar to the 33-mer peptide.

Food allergies to wheat also are complex. In a survey of 60 patients, Battais et al. (2005a) demonstrated that sera from allergy patients reacted with gluten proteins in all of the major groups and that the observed reactivity correlated with both the age and the symptoms of the patient. Using overlapping synthetic peptides, they identified IgE binding epitopes in alpha, gamma and omega gliadins and found that epitopes in food allergy were different from those in CD (Battais et al., 2005b).

A better understanding of the relationships between specific gluten proteins and their contributions to human health conditions and end-use functional properties is important for efforts to develop wheat that will be less likely to trigger immunogenic responses or better tolerated by patients with CD and food allergies. If the most highly immunogenic proteins could be eliminated from wheat flour without jeopardizing the functional properties of the flour, the introduction of that wheat into the marketplace may make it possible to reduce the numbers of people that become sensitized to wheat in the future. Alternately, making wheat flour safe for patients who already have CD or food allergy would require that all immunogenic proteins be eliminated or substantially reduced in the flour. This would include gluten proteins in all of the major groups and would likely impact the functional properties of the flour unless only those proteins within each group that contain harmful epitopes are identified and targeted.

This study focuses on the omega-1,2 gliadins, a subgroup of omega gliadins that are highly immunogenic. Omega gliadins are unusual even among gluten proteins in that they consist almost entirely of repetitive motifs with only short regions of unique sequence at their N- and C-terminal ends. The two types of omega gliadins, referred to as omega-1,2 gliadins and omega-5 gliadins, differ in N-terminal sequences and repetitive motifs. The omega-1,2 gliadins begin with ARE, ARQ, or KEL and contain the repetitive motif PQQPFP, while the omega-5 gliadins usually begin with the N-terminal sequence SRL and contain multiple copies of FPQQQ and QQIPQQ. The omega-1,2 gliadins are important in CD and in allergy patients that show a reaction to hydrolyzed wheat proteins (HWPs) in food products and cosmetics (Denery-Papini et al., 2012), while the omega-5 gliadins are the major sensitizing allergens in wheat-dependent exercise-induced anaphylaxis (WDEIA), a serious food allergy that occurs in sensitized individuals when the ingestion of wheat is followed by physical exercise (Morita et al., 2003).

In previous studies, we used RNAi to reduce the levels of omega-5 gliadins in wheat flour, resulting in transgenic plants with reduced IgE reactivity to sera from WDEIA patients without adverse effects on flour end-use quality (Altenbach and Allen, 2011; Altenbach et al., 2014b, 2015). The transgenic plants also had more stable protein compositions when produced under different levels of post-anthesis fertilizer. In this study, our goal was to design an RNAi construct that would target only the omega-1,2 gliadins in hopes of reducing the levels of immunodominant CD epitopes in wheat flour.

## Materials and Methods

### Plant Material

The United States hard red spring wheat *Triticum aestivum* ‘Butte 86’ was grown in a greenhouse with daytime/nighttime temperatures of 24/17°C as described previously (Altenbach et al., 2003). Plants were watered by drip irrigation with 0.6 g/l of Peters Professional 20-20-20 water-soluble fertilizer (Scotts-Sierra Horticultural Products Company, Marysville, OH, United States).

### RNA Interference Construct and Transformation of Plants

A 141 bp fragment from the 5′ end of an omega-1,2 gliadin gene was selected as the trigger for the RNAi construct. This fragment was amplified from 20 DPA endosperm RNA using primers QF18 and QR18 described in Altenbach and Kothari (2007), inserted in opposite orientations on either side of a 146 bp intron from a wheat starch synthase gene, then placed under the regulatory control of the HMW-GS Dy10 promoter and the HMW-GS Dx5 terminator as described in Altenbach and Allen (2011). The final construct was verified by DNA sequencing. Transformation of wheat plants with the construct and the plasmid pAHC25 that facilitates selection of transgenic plants with phosphinothricin (Christensen and Quail, 1996) was as described in detail in Altenbach and Allen (2011). Identification of putative transgenic plants by PCR analysis and initial screening of grain proteins from transgenic lines by SDS-PAGE were described previously (Altenbach and Allen, 2011). Homozygous lines were selected for transgenic plants in which the omega-1,2 gliadins were specifically eliminated from the grain without significant changes on other gluten proteins or where omega-1,2 gliadins as well as other gliadins and LMW-GS were eliminated from the grain.

### Protein Extraction and Analysis by Two-Dimensional Gel Electrophoresis (2-DE)

Grain from selected lines was pulverized into a fine powder and sifted sequentially through #25, 35, and 60 mesh screens. Total proteins were extracted from the resulting flour with SDS buffer (2% SDS, 10% glycerol, 50 mM DTT, 40 mM Tris-Cl, pH 6.8) and quantified using a modified Lowry assay as described in Dupont et al. (2011). Three separate extractions of flour were each analyzed three times by 2-DE as described in detail previously (Dupont et al., 2011). Gels were digitized using a calibrated scanner and analyzed using Progenesis SameSpots Version 5.0 (TotalLab, Ltd., Newcastle upon Tyne, United Kingdom). Identifications of individual protein spots in the Butte 86 non-transgenic line were reported in Dupont et al. (2011). Individual spots in transgenic lines were deemed to show significant changes from the non-transgenic if they had ANOVA *p*-values < 0.02 and had changes in average normalized spot volumes that were greater than 20%.

### Identification of Proteins in 2-DE Spots by Mass Spectrometry

The identities of proteins in selected 2-DE spots were confirmed by MS/MS. Protein spots #1–6 from Butte 86, #1–3 from transgenic line SA-30-118a-5 and #7–15 from transgenic line SA-30-118b-3 were excised from triplicate 2-D gels, placed in 96-well plates and digested individually with chymotrypsin, thermolysin, or trypsin using a DigestPro (Intavis, Koeln, Germany). Protein spots #1–6 from SA-30-118b-3 were digested with only trypsin. The resulting samples were then analyzed using an Orbitrap Elite mass spectrometer (Thermo Scientific, San Jose, CA, United States) as described in detail in Vensel et al. (2014). For analysis of spectral data, two search engines, Mascot^1^ and XTandem!^2^, were used to interrogate a database of 125,400 sequences that included Triticeae sequences downloaded from NCBI on 06-18-2018, gluten protein sequences from Chinese Spring reported by Huo et al. (2018a,b), Butte 86 sequences from Dupont et al. (2011) and Altenbach et al. (2011), Xioayan 81 gliadin sequences from Wang et al. (2017), and common MS contaminant sequences contained in the common Repository of Adventitious Proteins (cRAP)^3^. Data from the two searches and the three enzyme digestions were compiled and further validated using Scaffold version 4.7.5^4^ with a protein threshold of 99%, peptide threshold of 95% with 20 ppm error, and a minimum of 4 peptides. The decoy false discovery rate (FDR) in the analysis was 0%.

### Patients

Serum samples were from 20 CD patients with elevated levels of IgG antibody to gluten [14 female, 17 white race, mean (SD) age 40.7 (18.5) years] and 20 CD patients with elevated levels of IgA antibody to gluten [13 female, 19 white race, mean (SD) age 46.0 (17.3) years]. Positivity for IgG or IgA antibody reactivity to gluten was determined as described previously (Samaroo et al., 2010). All cases of CD were positive for antibody reactivity to transglutaminase 2 (the most sensitive and specific serologic marker of CD), determined as previously described (Lau et al., 2013). In addition, all patients were biopsy-proven, diagnosed with CD according to previously described criteria (Alaedini and Green, 2005), and on a gluten-containing diet. Serum samples were obtained under institutional review board-approved protocols at Columbia University. This study was approved by the Institutional Review Board of Columbia University Medical Center. All serum samples were maintained at -80°C to maintain stability.

### Assessment of Immune Reactivity by ELISA and 2-D Immunoblotting

Serum IgG and IgA antibody reactivities to gluten were measured separately by enzyme-linked immunosorbent assay (ELISA) as described previously (Moeller et al., 2014; Uhde et al., 2016), with some modifications. Gluten proteins were extracted from the non-transgenic Butte 86 and transgenic lines as described before (Huebener et al., 2015). A 2 mg/mL stock solution of the gluten extract in 70% ethanol was prepared. Wells of 96-well Maxisorp round-bottom polystyrene plates (Nunc, Roskilde, Denmark) were coated with 50 μL/well of a 0.01 mg/mL solution of protein extract in 0.1 M carbonate buffer (pH 9.6) or left uncoated to serve as controls. After incubation at 37°C for 1 h, all wells were washed and blocked by incubation with 1% bovine serum albumin (BSA) in PBS containing 0.05% Tween-20 (PBST) for 1.5 h at room temperature. Serum samples were diluted at 1:200 for IgA and at 1:800 for IgG measurement, added at 50 μL/well in duplicates, and incubated for 1 h. Each plate contained a positive control sample from a patient with biopsy-proven CD and elevated IgG and IgA antibodies to gluten. After washing, the wells were incubated with HRP-conjugated anti-human IgG (GE Healthcare, Piscataway, NJ, United States) or IgA (MP Biomedicals, Santa Ana, CA, United States) secondary antibodies for 50 min. The plates were washed and 50 μL of developing solution, containing 27 mM citric acid, 50 mM Na_2_HPO_4_, 5.5 mM *o*-phenylenediamine, and 0.01% H_2_O_2_ (pH 5), was added to each well. After incubating the plates at room temperature for 20 min, absorbance was measured at 450 nm. All serum samples were tested in duplicate. Absorbance values were corrected for non-specific binding by subtraction of the mean absorbance of the associated uncoated wells. The corrected values were normalized according to the mean value of the positive control duplicate on each plate.

Two-dimensional immunoblotting was also used to assess reactivity. Following 2-DE as described above, proteins were transferred onto nitrocellulose membranes using the iBlot Dry Blotting System (Life Technologies, Carlsbad, CA, United States). The membranes were incubated for 1 h in a blocking solution made of 5% milk and 0.5% BSA in a solution of Tris-buffered saline containing 0.05% Tween-20 (TBST). Incubation with patient serum specimens (1:2000 for IgA and 1:4000 for IgG determination in dilution buffer containing 10% blocking solution and 10% fetal bovine serum in TBST) was done for 1 h. Serum samples from representative patients with elevated IgA and/or IgG antibody reactivity to gluten were included. HRP-conjugated anti-human IgA and IgG were used as secondary antibodies. Detection of bound antibodies was by the ECL system (Millipore, Billerica, MA, United States) and the FluorChem M imaging system (ProteinSimple, San Jose, CA, United States). Following immunodetection, bound antibodies were removed from the nitrocellulose membranes with Restore Western blot stripping buffer (Thermo Scientific, Rockford, IL, United States) and the membrane proteins were visualized using colloidal gold stain (Bio-Rad, Hercules, CA, United States). Each immunoblot was aligned to its corresponding colloidal gold-stained membrane using the SameSpots software (version 5.0) (TotalLab Ltd., Newcastle upon Tyne, United Kingdom).

### Analysis of Flour End-Use Quality

End-use functionality tests were conducted at the USDA-ARS-HWWQL (Manhattan, KS, United States) using methods approved by American Association of Cereal Chemists International (AACCI, 1961, 1985, 1988, 1995) that are routinely used for assessment of wheat breeding lines. Wheat was converted to straight grade flour using a Quadramat Senior experimental flour mill following AACCI Method 26-10.02. Flour protein and moisture contents (14%mb) were determined by near-infrared reflectance (NIR) using AACCI method 39-11.01; mixing properties were determined on 10 g (14%mb) flour samples using a Mixograph (TMCO, National Mfg., Lincoln, NE, United States) according to AACCI Method 54-40.02; and SDS sedimentation tests were conducted in adherence to AACCI Method 56-60.01. Averages and standard deviations from triplicate samples were calculated for the non-transgenic and SA-30-118a-5 transgenic lines.

## Results

### Selection of a Target Region for the RNAi Construct

**FIGURE 1:**
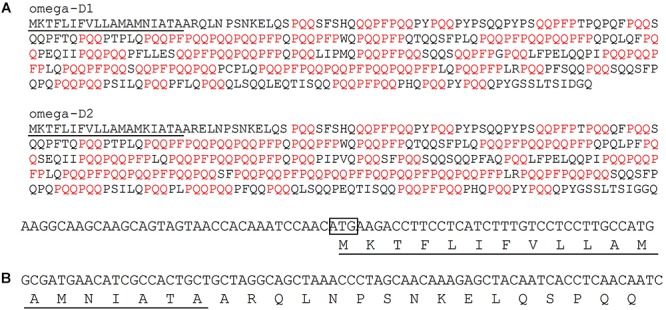
Design of the target region for the RNAi construct. **(A)** Sequences of two omega-1,2 gliadins from Chinese Spring with PQQ and QQPFP motifs shown in red. The signal peptide is underlined. **(B)** Nucleotide sequence of the 141 bp target region used in the RNAi construct and the portion of the protein encoded by the target region. In the DNA sequence, the initiation codon is enclosed in a box. In the protein sequence, the signal peptide is underlined.

The sequences of two omega-1,2 gliadins, omega-D1 and omega-D2, whose genes were identified from Chinese Spring by Huo et al. (2018b) are shown in Figure 1A. These proteins contain ∼70% proline + glutamine and have central regions that contain 20 QQPFP and either 45 or 53 PQQ motifs, respectively. These motifs are also found in other gliadins and LMW-GS and are present within the sequences of several characterized CD epitopes (Supplementary File 1). For example, 21 of 26 alpha gliadins, all delta gliadins and three of 10 LMW-GS from Chinese Spring contain a QQPFP motif while the 11 gamma gliadins contain from two to nine copies of the sequence. Multiple copies of the PQQ motif are also found in all other gliadins and LMW-GS from Chinese Spring. To design a RNAi construct specific for omega-1,2 gliadin genes, a 141 bp region that included 34 bp of the 5′ untranslated region as well as the portion of the gene encoding the signal peptide and N-terminal region of the protein was selected as the trigger sequence (Figure 1B). The specificity and potential off-target effects of the construct were assessed by comparing the trigger sequence to the genomic regions containing gluten protein genes from chromosomes 1A, 1B, 1D, 6A, 6B, and 6D from Chinese Spring (NCBI Accessions MG560140, MG560141, MG560142, MH338176, MH338181, and MH338193, respectively) (Huo et al., 2018a,b). Of the full-length omega-1,2 gliadin genes from Chinese Spring, the target region had 30 and 110 bp regions of identity with the omega-D1 gene and 20, 33, and 44 bp regions of identity with the identical omega-D2 and -D3 genes. In addition, there were multiple regions of identity that ranged from 20 to 90 bp with pseudogenes omega-A1, -A2, -A3, and -A4. Of the other expressed gluten protein genes described by Huo et al. (2018a,b), only seven contained regions of identity greater than 20 bp. These were the omega-5 gliadin genes, omega-B3 and -B6, containing 34 bp regions of identity, and LMW-GS genes, LMW-B2, -B3, -D1, -D6, and -D8, containing 23 bp regions of identity. Identities were within the portions of the genes encoding signal peptides.

### Analysis of Flour Proteins From Transgenic Lines

**FIGURE 2:**
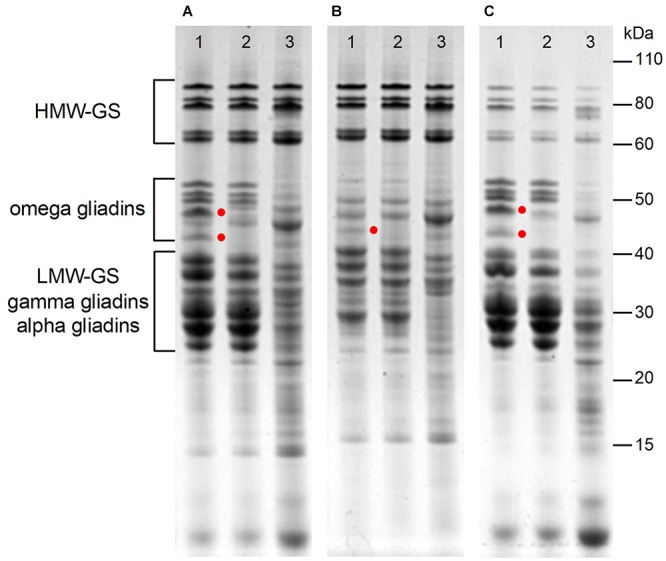
Analysis of total protein **(A)**, glutenin **(B)**, and gliadin **(C)** in flour of non-transgenic (lane 1), and transgenic lines 118a-5 (lane 2) and 118b-3 (lanes 3). Red dots highlight bands that are present in the non-transgenic and absent in 118a-5.

Following transformation of Butte 86 plants with the RNAi construct, transgenic plants were identified that contained grain with altered protein profiles. Figure 2 shows total protein, glutenin, and gliadin fractions from the grain of two transgenic lines that were selected for detailed analysis. In line SA-30-118a-5, referred to as 118a-5, several bands between 40 and 50 kDa were missing in the total protein, glutenin and gliadin fractions (Figure 2A–C, lane 2), but most other gluten proteins were not affected. In contrast, line SA-30-118b-3, referred to as 118b-3, showed notable changes in all protein fractions (Figure 2A–C, lane 3).

**FIGURE 3:**
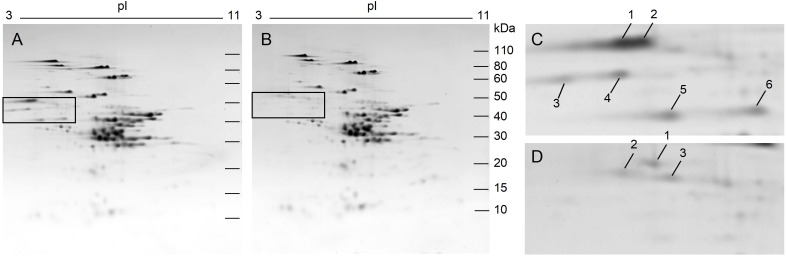
2-DE analysis of total flour proteins from non-transgenic **(A)** and transgenic plant 118a-5 **(B)**. The black boxes in **(A,B)** show the positions of the omega-1,2 gliadins. Regions of the gels containing omega-1,2 gliadins are enlarged in **(C)** (non-transgenic) and **(D)** (transgenic). Spots that are numbered were identified by MS/MS.

Analysis of total proteins from the non-transgenic control and 118a-5 by 2-DE is shown in Figure 3. Major differences between the two lines are highlighted in the boxes in Figure 3A,B. Although the positions of these spots in 2-DE were consistent with omega-1,2 gliadins identified by Dupont et al. (2011), the identifications were confirmed by MS/MS in the non-transgenic line (Supplementary File 2 and Figure 3C). Spots 1 and 2 contained omega-1,2 gliadin BAN29067 as well as protein disulfide isomerase and beta-amylase. BAN29067 is identical to omega-D2 and -D3 from Chinese Spring except that it is missing the signal peptide. Spot 3 was identified as omega-1,2 gliadins BAN29067 and CAR82265 while spot 4 was identified as CAR82265. CAR82265 is the same as omega-D1 from Chinese Spring except for an extra amino acid at the N-terminus. This omega-1,2 gliadin contains a single cysteine residue. Spots 5 and 6 contained omega-1,2 gliadin AKB95614. ABK95614 is similar to the protein that would be encoded by pseudogene omega-A1 from Chinese Spring if the stop codon midway through the coding region was removed (Supplementary File 2). These spots were absent in 118a-5. Several minor spots from the transgenic line that appeared in the omega-1,2 gliadin region of the gel were also identified. Spot 1 in Figure 3D contained protein disulfide isomerase and beta-amylase while spots 2 and 3 were identified as beta-amylases. Quantitative 2-DE analyses revealed that there were few changes in the levels of individual proteins other than the omega-1,2 gliadins in the transgenic line (Table 1 and Supplementary File 3). Gluten proteins accounted for 73.9% of the total normalized spot volume in the non-transgenic lines and 71.2% in the transgenic line. Gliadins accounted for 40.3% of the spot volume in the non-transgenic and 36.4% in 118a-5 while the proportions of glutenins were similar in both lines (Supplementary File 3).

**Table 1:** Changes in the amounts of different classes of flour proteins in transgenic lines relative to the non-transgenic.

	% Change	
	118a-5	118b-3
Alpha gliadins	5.8	-58.9
Gamma gliadins	-7.1	-73.1
Omega-1,2 gliadins	-68.6	-74.6
Omega-5 gliadins	-10.1	-85.1
HMW-GS	7.6	27.1
LMW-GS	-6.6	-61.0
Purinins	16.5	150.9
Farinins	9.5	60.2
Triticins	11.8	108.1
Globulins	0.6	77.8
Serpins	11.3	51.4
AAI	8.8	90.5
Other non-gluten proteins	-1.4	58.9

Total gliadins	-12.7	-70.1
Total glutenins	0.0	-17.7
Total gluten proteins	-6.9	-45.7
Total non-gluten proteins	6.9	80.0


**FIGURE 4:**
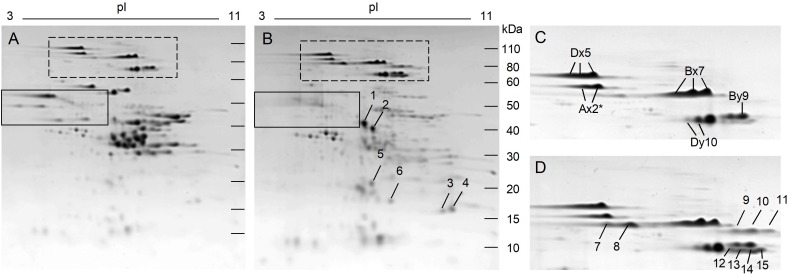
2-DE analysis of total flour proteins from non-transgenic **(A)** and transgenic plant 118b-3 **(B)**. The solid black boxes in **(A,B)** show the positions of the omega-1,2 gliadins while the dashed boxes show the positions of the HMW-GS. Regions of the gels containing HMW-GS are enlarged in **(C)** (non-transgenic) and **(D)** (transgenic). Arrows in **(B,D)** point to spots that increased in the transgenic line and were identified by MS/MS.

In contrast, changes in the protein profile of transgenic line 118b-3 relative to the non-transgenic were quite dramatic (Figure 4). In addition to the omega-1,2 gliadins shown in the black box, nearly all gliadins and LMW-GS were suppressed (Figure 4B). A number of other proteins showed obvious increases in this line and were identified by MS/MS. Spots 1-4 in Figure 4B were identified as triticins, proteins similar to 11S storage proteins from dicots that have a large subunit and a small subunit cleaved from a larger precursor. Spots 1 and 2 in Figure 4B correspond to the large subunit encoded by the 5′ portions of the genes while spots 3 and 4 correspond to the small subunit encoded by the 3′ portions of the genes (Supplementary File 4). Spot 5 was identified as a purinin and spot 6 was identified as the endogenous alpha amylase/subtilisin inhibitor referred to as WASI. In addition, a number of spots that were either minor or undetectable in the HMW-GS region of the non-transgenic line increased in 118b-3 (Figure 4B, dashed box; 4D). Spots 7 and 8 in Figure 4D were identified by MS/MS as HMW-GS Ax2^∗^, spots 10 and 11 as HMW-GS Bx7 and spots 12–15 as HMW-GS By9, consistent with identifications obtained from Butte 86 in a previous report by Dupont et al. (2011) (Supplementary File 4). Another transgenic line, SA-30-131b-5, showed a similar 2-DE profile. From the quantitative 2-DE analysis, HMW-GS accounted for 17% of the total protein in the non-transgenic, but 25% of the total protein in 118b-3 (Supplementary File 5). Interestingly, most of the increase was due to HMW-GS spots 7–15. All other gluten proteins accounted for 57.3% of the total protein in the non-transgenic, but only 21.6% of the protein in the transgenic (Supplementary File 5). The decline in total gluten protein in the transgenic was compensated by an increase in the non-gluten proteins from 25.7 to 53.5% of the total protein. Indeed, there were significant increases in most proteins within the major groups of non-gluten proteins, including triticins (108.1%), purinins (150.9%), farinins (60.2%), globulins (77.8%), serpins (51.4%), and AAI (90.5%) (Table 1).

### Immunogenic Potential of Transgenic Lines

**FIGURE 5:**
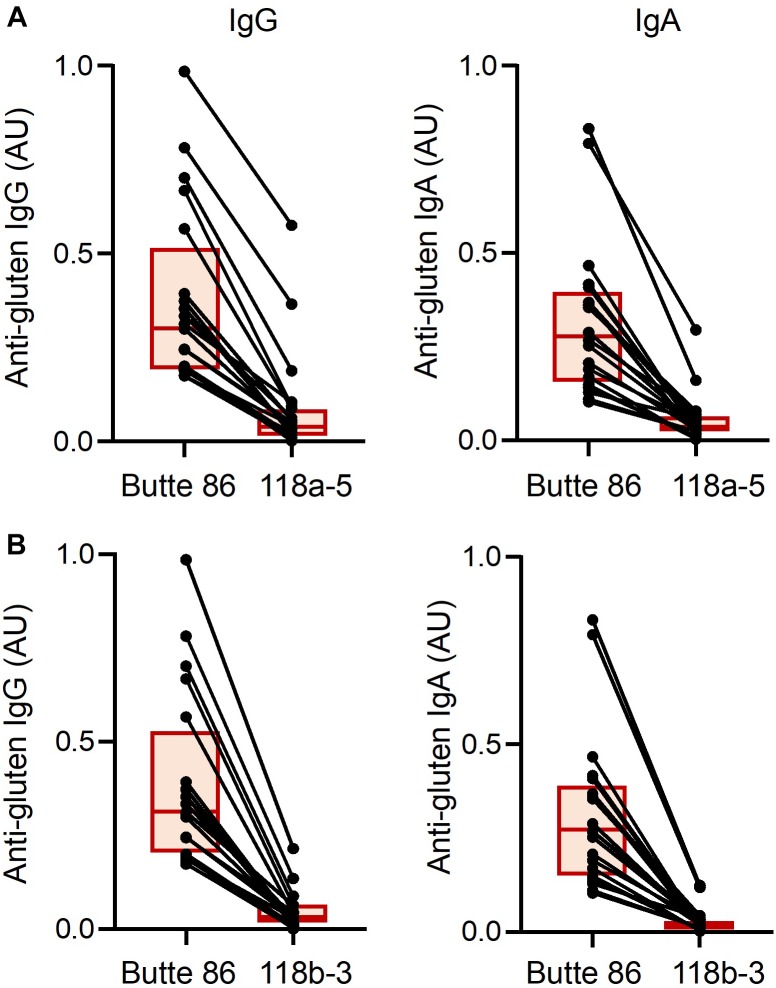
Levels of IgG and IgA antibody reactivity for each of the 20 anti-gluten IgG-positive and 20 anti-gluten IgA-positive celiac disease patients toward gluten proteins from non-transgenic and transgenic plants 118a-5 **(A)** and 118b-3 **(B)**. Each individual is represented by a dot and the two points corresponding to the same individual are connected by a line. Each box indicates the 25th–75th percentiles of distribution, with the horizontal line inside the box representing the median.

**FIGURE 6:**
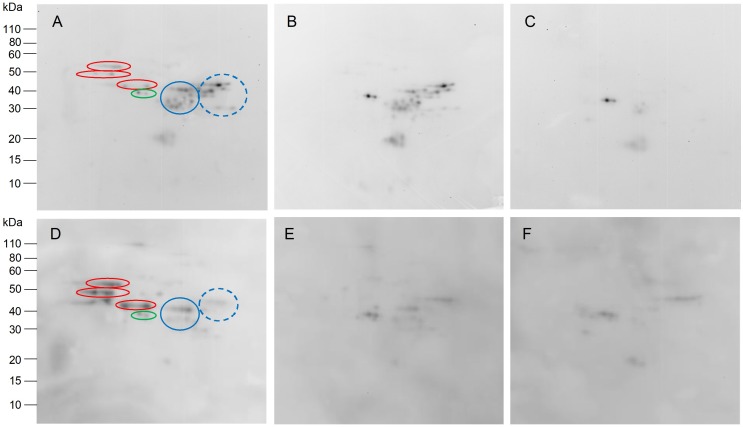
Immunoblots showing IgG **(A–C)** and IgA **(D–F)** antibody reactivity in two representative celiac disease patients toward two-dimensionally separated total flour proteins from non-transgenic **(A,D)** and transgenic plants 118a-5 **(B,E)** and 118b-3 **(C,F)**. Omega-1,2 gliadins in **(A,D)** are shown in red ovals. Alpha and gamma gliadins are shown in blue circles, LMW-GS in blue dashed circles and serpins in green ovals.

Reactivities of serum IgG and IgA antibodies from biopsy-proven CD patients toward gluten proteins were examined in the non-transgenic and transgenic wheat lines. Levels of detected IgG and IgA antibodies were highly diminished in the transgenic lines when compared to the non-transgenic line as determined by ELISA (*p* < 0.0001 for all comparisons) (Figure 5). All patients in the study had lower IgG and IgA reactivities to 118b-3 than to 118a-5, although differences were small for many patients. The molecular specificity of the reduction in CD antibody binding to gluten proteins was further examined by two-dimensional immunoblotting (Figure 6). For the representative patient shown in Figure 6A, IgG serum antibodies reacted with omega-1,2 gliadins, alpha and gamma gliadins, LMW-GS and serpins. Reactivity to omega-1,2 gliadins was eliminated in 118a-5 while reactivity to all gluten proteins was eliminated in 118b-3. IgA serum antibodies from the patient shown in Figure 6D showed the greatest reactivity to the omega-1,2 gliadins. This reactivity was eliminated in both 118a-5 and 118b-3.

### End-Use Quality of Flour From Transgenic Lines

**FIGURE 7:**
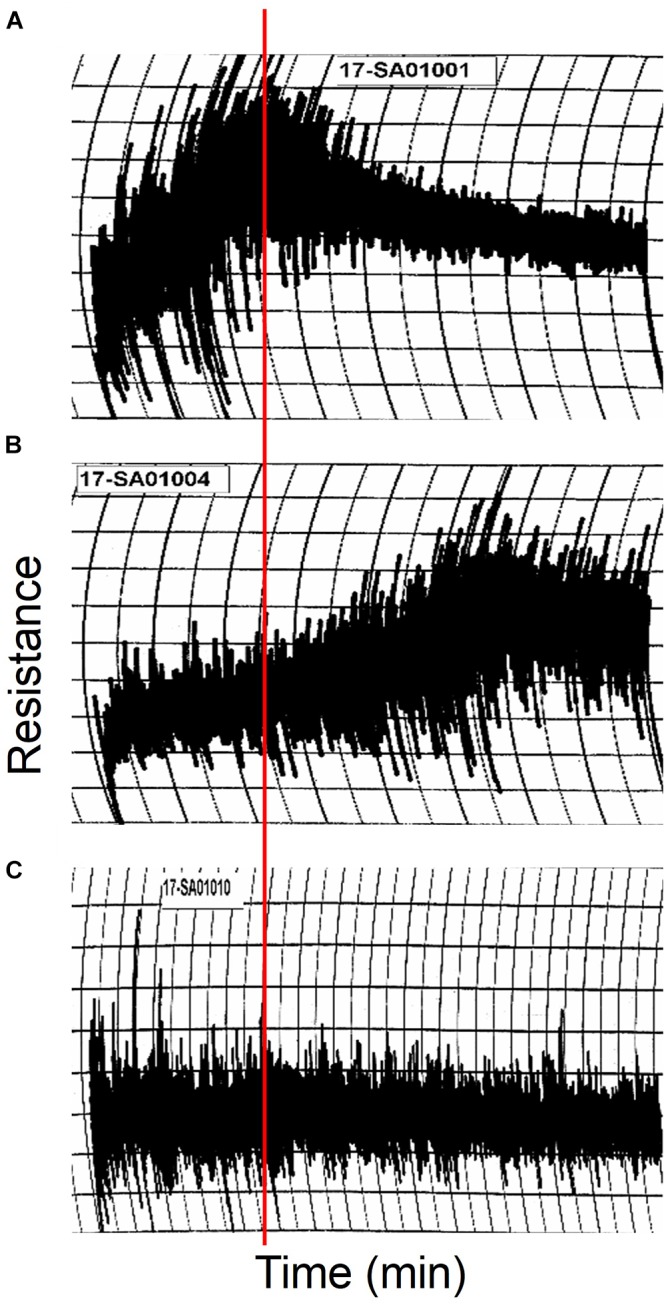
10 g mixogram curves produced using flour from non-transgenic **(A)** and transgenic lines 118a-5 **(B)** and 118b-3 **(C)**. The red line shows the position of the peak mixing time in the non-transgenic.

Non-transgenic and transgenic lines were grown in the greenhouse in sufficient quantities for end-use quality testing. The resulting grain from all lines had a vitreous appearance. Average kernel weight of 118a-5 was similar to that of the non-transgenic, 46.2 ± 1.4 mg for 118a-5 vs. 49.3 ± 2.6 mg for the non-transgenic. Grain protein and flour protein percentages also were similar for the two lines (Table 2). However, there were notable differences in the 10 g mixogram curves for each of the two lines (Figure 7A,B). Mix time was increased from 2.5 min for flour from the non-transgenic to 5.8 min for flour from 118a-5. In addition, mixing tolerance increased from 2 in the non-transgenic to 6 in 118a-5. There was also an increase in the SDS sedimentation volume from 62.8 ml in the non-transgenic to 66.1 ml in 118a-5 (Table 2).

**Table 2:** End-use quality data from non-transgenic and transgenic lines.

	Grain protein (%)	Flour protein^1^ (%)	Mix time (min)	Mix Tolerance^2^	SDS sedimentation volume (ml)
Butte 86^3^	20.5 (0.8)	18.3 (0.5)	2.5 (0.1)	2	62.8 (0.45)
118a-5^3^	20.2 (0.9)	17.8 (0.8)	5.8 (0.1)	6	66.1 (0.87)


*^1^Based on 14% moisture. ^2^Recorded on a 0–6 scale with 6 having the greatest tolerance. ^3^Averages and (standard deviations) from flour samples from three biological replicates are reported*.

In comparison, the average kernel weight was reduced ∼24% for 118b-3 and grain and flour protein percentages were 17.8 and 12.9% less, respectively, in 118b-3 than in the non-transgenic. The mixogram curve from 118b-3 flour was essentially flat, making it difficult to determine accurate mix times and tolerances (Figure 7C). The SDS sedimentation volume of 33.6 ml also was ∼46% less than the non-transgenic.

## Discussion

With the exception of short regions at the N- and C-termini, the omega-1,2 gliadins consist entirely of repetitive sequences that are also found in other gliadins and some LMW-GS. Careful selection of a 141 bp trigger from the 5′ region of the omega-1,2 gliadin gene for the RNAi construct made it possible to silence only the genes of interest in transgenic line 118a-5. Surprisingly, transformation with the same construct also resulted in transgenic lines in which nearly all gliadin and LMW-GS genes were suppressed. Currently, full-length sequences of only 13 alpha gliadin, 9 gamma gliadin and 5 LMW-GS genes from cv. Butte 86 are available (Altenbach et al., 2010a,b; Dupont et al., 2011). However, comparison of the RNAi target with a complete set of gluten protein gene sequences from Chinese Spring revealed that only omega-5 gliadin and a few LMW-GS genes had regions of identity with the trigger that were greater than 20 bp. Nonetheless, alpha and gamma gliadins were down-regulated effectively by the construct in some plants. As reviewed by Senthil-Kumar and Mysore (2011), there are many other factors that could contribute to off-target silencing, including the size of the trigger, the region of the gene that it targets and the specificity of the promoter that is used for expression. In addition, the site of integration in the genome and the copy number of the insertion can influence off-target effects.

Compensatory effects on the proteome were also noted in 118b-3 but not in 118a-5. Little is known about how the wheat grain compensates for alterations in different groups of storage proteins. The omega-1,2 gliadins encompass only about 6.6% of the total flour protein in Butte 86. In 118a-5, it is likely that any compensation for the loss of these proteins was spread over the entire proteome. In contrast, gliadins and LMW-GS account for ∼58% of the total flour protein in Butte 86 and the large decreases in these proteins in 118b-3 were compensated by obvious increases in HMW-GS as well as in many groups of non-gluten proteins including triticins, serpins, purinins, globulins, and AAI. Among the HMW-GS, it is particularly curious that several protein spots that migrate faster than the major Ax2^∗^, By9, and Bx7 spots in the second dimension of 2-DE were more prominent in the transgenic line. Differences between the apparent molecular weights of these protein spots and the major HMW-GS spots in the non-transgenic suggest that these spots could result from post-translational modifications. Recently, Nunes-Miranda et al. (2017) presented evidence that y-type HMW-GS encoded by the B genome are subject to proteolytic processing at the C-terminus by an asparaginyl endopeptidase. This processing event likely occurs at one of two asparagine residues 36 and 42 amino acids upstream of the C-terminus of the protein, resulting in the removal of a cysteine residue that may be involved in the formation of the glutenin polymer. Thus, the processed HMW-GS potentially could influence glutenin polymer size and end-use quality. Certain omega-1,2 gliadins and some LMW-GS have also been shown to undergo post-translational cleavage at the N-terminus by an asparaginyl endopeptidase (Dupont et al., 2004; Egidi et al., 2014). In the absence of the omega-1,2 gliadins and LMW-GS in 118b-3, it is possible that HMW-GS By9 may be a preferred substrate for the enzyme. While this explanation seems plausible, it is not supported by the MS/MS data that identified a number of peptides within 36 amino acids of the C-termini of the proteins in spots 12-15 (Figure 4D and Supplementary File 4). It is also notable that HMW-GS Ax2^∗^ and Bx7 do not contain any asparagine. However, asparaginyl endopeptidases are reported to cleave at aspartate residues with a lower efficiency and these residues are found in both proteins (Müntz et al., 2002). But, as with By9, C-terminal peptides (in spots 7 and 11) or peptides close to the C-termini (in spots 8 and 10) were identified by MS/MS.

Off-target and compensatory effects due to RNAi silencing of wheat gluten protein genes have also been observed in a number of studies. In RNAi experiments targeting the omega-5 gliadins, Altenbach et al. (2014a) reported differential effects on the proteome, although it was possible to identify transgenic plants in which only the omega-5 gliadins were reduced. Barro et al. (2015) evaluated seven different combinations of RNAi constructs with the goal of creating a wheat line that was devoid of CD epitopes and observed a wide variety of effects. Their constructs included target regions derived from both repetitive and non-repetitive portions of alpha, gamma and omega gliadin and LMW-GS genes. In some constructs, target regions from multiple genes were combined. Additionally, plants were sometimes transformed with more than one RNAi construct. In plants transformed with an RNAi construct targeting a 169 bp region corresponding to the Q-rich domain of a gamma gliadin, gamma gliadins decreased but were compensated by increases in omega gliadins (Gil-Humanes et al., 2010). Likewise, when a 377 bp region corresponding to the repetitive domain from an alpha gliadin was used as the target, alpha gliadins decreased while omega gliadins and HMW-GS increased. In one construct, a 132 bp region that encoded part of the signal peptide, N-terminal region and the repetitive domain of a LMW-GS was combined with a 173 bp region encoding a repetitive region from an omega gliadin. In the two resulting transgenic lines, LMW-GS decreased, omega, gamma and alpha gliadins were partially decreased and HMW-GS increased. In another construct, a 170 bp region that corresponded to the first non-repetitive domain of an alpha gliadin was combined with a 191 bp region that corresponded to part of the signal peptide, N-terminal region and repetitive region of an omega gliadin. Both were from the most conserved regions of the genes. In the three lines reported, omega and gamma gliadins decreased, alpha gliadins were partially decreased, HMW-GS increased and LMW-GS were somewhat increased. Other combinations of RNAi plasmids resulted in transgenic plants with decreased CD toxicity. In plants transformed with two RNAi constructs, one targeting alpha gliadins and the other targeting both LMW-GS and omega gliadins, all gliadins and LMW-GS decreased while HMW-GS increased. These results are similar to those obtained the current study in 118b-3 using a construct that contained target sequences from only an omega-1,2 gliadin. Barro et al. (2015) did not evaluate constructs containing sequences derived only from omega gliadins. Additionally, they evaluated effects on the proteome by HPLC rather than 2-DE so it is not known whether increases in HMW-GS observed in their lines might reflect post-translational processing.

With regard to immunogenicity, the analyses with antibodies from CD patients demonstrated a highly significant reduction in binding to the transgenic lines when compared to the non-transgenic wheat. The data suggest that removal of the omega-1,2 gliadins is likely to have a considerable effect in eliminating the pathogenic sequences present in the studied Butte 86 wheat cultivar. Whether a similarly significant reduction in T cell reactivity to the transgenic lines would be observed remains to be seen in further analyses. However, considering the fact that the omega-1,2 gliadins contain known T cell epitopes and that most T cell epitopes are located within B cell epitope sequences of gluten proteins, it is highly likely that T cell reactivity to the transgenic lines would also be eliminated or diminished to a great extent.

With regard to flour quality, removal of the omega-1,2 gliadins from the flour resulted in flour with increased mix times and tolerances. There was also a small increase in SDS sedimentation volume in the absence of these proteins. Taken together, this suggests that the omega-1,2 gliadins have a negative effect on flour mixing properties. It should also be noted that the omega-1,2 gliadins in spots 3 and 4 in Figure 3C have been shown previously to be preferentially accumulated in small glutenin polymer fractions (Vensel et al., 2014). The omega-1,2 gliadins identified in these spots by MS/MS contain a single cysteine residue and likely function as chain terminators of the polymer. These proteins constitute about 20% of the omega-1,2 gliadins in Butte 86 and would be expected to reduce the size of the polymers and decrease dough strength. In similar studies using RNAi, omega-5 gliadins were also shown to have a negative effect on end-use quality. However, the omega-5 gliadins in Butte 86 do not contain any cysteine and are not associated with glutenin polymers (Altenbach et al., 2014b).

The mixing properties of flour from 118b-3 were diminished as evidenced by the flat mixing curve and the reduction in SDS sedimentation volumes. Perhaps this is not surprising given the absence of most gliadins and LMW-GS. Flat mixing curves were also observed by Gil-Humanes et al. (2014b) using a 35 g mixograph for transgenic lines with reduced levels of gliadins and, in some cases, LMW-GS. Many of their lines also showed reduced SDS sedimentation values. Nonetheless, Gil-Humanes et al. (2014a) demonstrated that the flour could be used to produce a reduced-gliadin bread that, while not optimal, was at least more acceptable than some of the current gluten-free bread options. Further studies are necessary to determine whether flour from 118b-3 could be used to produce a similar product. Alternately, flour from 118b-3 may prove useful as a base flour to test the effects of individual gluten protein components on flour functional properties. For example, specific types of LMW-GS or chain-terminating gliadins might be added in mixing studies to evaluate their roles on glutenin polymer formation. Additionally, different types and amounts of gliadins could be added to determine how the balance of gliadins and glutenins affects end-use quality. It may also be interesting to examine disulfide linkages between HMW-GS in transgenic line 118b-3 since most other gluten proteins are absent in this line.

While these studies provide new information about the role of specific gluten proteins in flour end-use quality and human health, an important question is whether these or similar lines could be of commercial value. Thus far, consumer attitudes have prevented the release of transgenic wheat in the marketplace both in the United States and abroad. Genome editing offers an alternative approach to RNAi and allows similar changes to be made to the flour proteome with the important advantage that the resulting plants are not considered transgenic and could be incorporated into conventional wheat breeding programs, at least in the United States. In some of the first genome editing studies, Sánchez-Léon et al. (2018) demonstrated success in using two single guide RNAs (sgRNAs) with homology to conserved regions of alpha gliadin genes to reduce both the levels of alpha gliadins and the immunoreactivity of the flour. Their study targeted the majority of alpha gliadin genes. However, the fact that only 20 bp of identical sequence are required for sgRNAs suggests that it should be possible to target individual gluten protein genes or small subgroups of genes provided that detailed sequence knowledge about the complete set of gluten protein genes in the wheat cultivar being modified is available. Given the complexity of the gluten proteins, the road ahead is not easy. The results nonetheless suggest that biotechnology approaches can be used in the future to improve the healthfulness of wheat, while maintaining or even improving its end-use qualities.

## Data Availability

This manuscript contains previously unpublished data. The name of the repository and accession number are not available.

## Author Contributions

SA designed the study, analyzed the data, and wrote the manuscript. AA contributed to study design, data analysis, and writing of the manuscript. BS was responsible for end-use quality testing and interpretation of results. H-CC conducted genetic transformation experiments, analyses of flour proteins by 2-DE and MS/MS analyses. XY was responsible for immunoblotting and ELISA experiments and interpretation of results. PG was responsible for subject recruitment and clinical characterization of patients. All authors contributed to editing of the manuscript and approved the manuscript.

## Conflict of Interest Statement

The authors declare that the research was conducted in the absence of any commercial or financial relationships that could be construed as a potential conflict of interest.

## Footnotes

1 www.matrixscience.com

2 https://www.thegpm.org/TANDEM/

3 ftp://ftp.thegpm.org/fasta/cRAP/

4 http://www.proteomesoftware.com/

## Abbreviations

2-DE: two-dimensional gel electrophoresis
AAI: alpha amylase/protease inhibitors
CD: celiac disease
HMW-GS: high molecular weight glutenin subunits
LMW-GS: low molecular weight glutenin subunits
MS/MS: tandem mass spectrometry
RNAi: RNA interference
